# Pre-emptive and therapeutic value of blocking bacterial attachment to the endothelial alphaVbeta3 integrin with cilengitide in sepsis

**DOI:** 10.1186/s13054-017-1838-3

**Published:** 2017-09-26

**Authors:** Carolina D. Garciarena, Tony M. McHale, Ignacio Martin-Loeches, Steve W. Kerrigan

**Affiliations:** 1 Cardiovascular Infection Research Group, Irish Centre for Vascular Biology, Royal College of Surgeons in Ireland, Dublin 2, Ireland; 2 Trinity Centre for Health Sciences, St. James University Hospital, Dublin 8, Ireland; 3 Cardiovascular Infection Research Group, Royal College of Surgeons in Ireland, 123 St. Stephen’s Green, Dublin 2, Ireland

Sepsis is a fast progressing disease, triggered by the host response to infection. If untreated, sepsis can rapidly evolve to multi-organ dysfunction and septic shock [1]. Despite numerous advances in palliative intensive care and antibiotic treatment, sepsis remains a major cause of morbidity and mortality [2]. The lack of effective therapies making it to market is due to our poor understanding of the early mechanisms driving sepsis.

The vascular endothelium is a major target of sepsis-induced events [2, 3]. Upon entry to the bloodstream, bacteria attach to the endothelium within 15 s [4]. Attachment triggers dysregulated signals that result in endothelial cell (EC) death and loss of barrier integrity, which give rise to increased capillary permeability clinically associated with hypotension, subcutaneous and body-cavity oedema and impaired tissue oxygenation, key events leading to multi-organ failure.

We have recently reported bacterial binding to the major EC integrin αVβ3 as a novel host-pathogen interaction that occurs early in sepsis [4, 5]. By this mechanism, both *Staphylococcus aureus* and *Escherichia coli* (primary triggers of sepsis) induce loss of junction protein VE-cadherin, which weakens the EC barrier and increases permeability. We identified that blocking αvβ3 with cilengitide prevents bacterial binding and attenuates EC injury [4, 5] when given prior to bacterial challenge. To ascertain if cilengitide would be useful post-bacterial attachment we perfused clinical strains of *S. aureus* or *E. coli* over human ECs for 360 s using a real time ex vivo model of sepsis. Cilengitide (0.05 μM) was either co-administered with the bacteria from t = 0 s (pre-emptive effect) or introduced to the suspension at t = 15, 30 and 180 s (therapeutic effect).

Our data demonstrate that *S. aureus* and *E. coli* binding to ECs increases steadily over time (Fig. 1). When applied at t = 0, cilengitide (0.05 μM) completely abolished *S. aureus* and *E. coli* attachment to ECs. Following bacterial attachment to ECs (at t = 15, 30 and 180 s), cilengitide significantly displaced bound bacteria in a time-dependent manner, rapidly reducing bacterial load back to background levels.

**Fig. 1:**
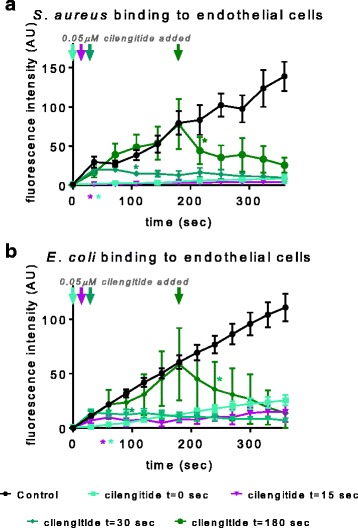
*S. aureus* (**a**) and *E. coli* (**b**) binding to EC monolayers under flow. *S. aureus* and *E. coli* were labelled with fluorescein. Bacterial suspensions were perfused through Ibidi chambers over confluent EC monolayers and timelapse bacterial binding was measured by fluorescence microscopy. Bacteria binding to EC was assessed in the presence or absence of cilengitide. Cilengitide was added at different time points (indicated by arrows): t = 0 s, t = 15 s, t = 30 s and t = 180 s (n = 3–6 for *S. aureus* and n = 3–11 for *E. coli*). *Asterisks* indicate that results are different from control form that point onwards, *P* < 0.05

These results suggest that cilengitide is capable of competitively antagonizing bacterial binding to ECs and as a result removes the signal that perpetuates vascular EC involvement in sepsis, and thus presents as a potential as new complementary strategy for the treatment of established sepsis and as prophylaxis in high risk patients. These observations warrant the initiation of preclinical and human clinical trials to validate the use of cilengitide as a pharmacological tool to reduce risk and/or increase the time window for decision-making in sepsis patients.

## Abbreviation

EC: Endothelial cell
